# Low-voltage electrical injuries and the electrocardiogram: is a ‘normal’ electrocardiogram sufficient for safe discharge from care? A systematic review

**DOI:** 10.29045/14784726.2023.12.8.3.27

**Published:** 2023-12-01

**Authors:** Simon Corrall, Samantha Laws, Alan Rice

**Affiliations:** Lymington Urgent Treatment Centre; St. George’s, University of London; North Shropshire PCN

**Keywords:** arrhythmia, electrical injury, electrocardiogram

## Abstract

**Introduction:**

The current Joint Royal Colleges Ambulance Liaison Committee guidelines in the United Kingdom provide clear national guidance for low-voltage electrical injury patients. While patients can be considered safe to discharge with an apparently ‘normal’ initial electrocardiogram (ECG), some evidence questions the safety profile of these patients with a risk of a ‘delayed arrhythmia’. This review aims to examine this as well as identifying the frequency and common arrhythmias that require patients to be conveyed to hospital for further monitoring post electrical injury. It will also aim to improve the understanding of potentially clinically significant arrhythmias that may require clinical intervention or even admission within an in-hospital environment.

**Methods:**

A systematic review using three electronic databases (CINAHL, MEDLINE, AMED) was conducted in January 2022. A preferred reporting items for systematic reviews and meta-analyses (PRISMA) approach was used to identify relevant studies with a suitable quality to support a critical review of the topic. A modified Critical Appraisal Skills Programme quality assessment checklist was used across suitable studies and a descriptive statistics approach was adopted to present the findings.

**Results:**

Seven studies, largely retrospective reviews, met the inclusion criteria. The findings showed 26% of patients had an arrhythmia on initial presentation (n = 364/1234) with incidences of sinus tachycardia, sinus bradycardia and premature ventricular contractions. However, making definitive statements is challenging due to the lack of access to individual patients’ past ECGs. Within these arrhythmias’ ST segment changes, atrial fibrillation and long QT syndrome could be considered potentially significant, however associated prognosis with these and electrical injuries is unknown. Only six (0.5%) patients required treatment by drug therapy, and a further three died from associated complications. Most patients with a normal ECG were discharged immediately with only a limited follow-up. No presentation of a ‘delayed arrhythmia’ was identified throughout the studies.

**Conclusion:**

The data for low-voltage electrical injuries are limited, but the potential arrhythmias for this patient group seldom require intervention. The entity of the ‘delayed arrhythmia’ may not be a reason to admit or monitor patients for prolonged periods. Further studies should consider the safety profile of discharging a patient with a normal ECG.

## Introduction

Electrical injuries are considered an uncommon, yet potentially devastating injury (Waldman & Combes, 2017). Much of the focus of the research surrounding electrical injuries remains on the higher-voltage causes (> 1000 V), as the resultant injury patterns lead to significant outcomes such as complex burns, fractures or cardiac arrest (Gentges & Schieche, 2018). However, while paramedics are aware that arrhythmias are a potential occurrence post electrical injury, and therefore recognise the importance of obtaining an electrocardiogram (ECG), the typical arrhythmias and prognosis of adult patients are less understood (Waldman & Combes, 2017). Within the United Kingdom, limited data are available about these patients attending acute healthcare services, as it is often thought that many patients will not seek assessment unless there are other factors involved such as burns, or the need for analgesia (Warentis et al., 2020).

Some authors have considered that low voltages can cause injury to the myocardium, and the typical flash or thermal burns may be regarded as a ‘distracting injury’ that may lead the clinician to miss or underestimate a potentially dangerous arrhythmia (Zemaitis et al., 2020). Single case reports have highlighted the possibility of potentially fatal arrhythmias such as ventricular fibrillation or ventricular tachycardia after electrocution from a low-voltage source (Guimaraes et al., 2020; Sharma et al., 1990). Furthermore, another perturbing outcome often associated with low-voltage injuries involves the theory of the ‘delayed arrhythmia’; a potentially fatal arrhythmia may occur days or even months after the initial insult. However, the most commonly referenced source on this topic is Jensen et al. (1987), which is a dated study based only on two low-voltage patient case reports, and arguably has considerable flaws in determining if the eventual arrhythmias were attributed to the initial electric shock.

Many recent studies such as Bailey et al. (2007) grouped the data from both low- and high-voltage patient group outcomes together, which may have contributed to the potential thinking that both groups have similar patient ECG features and prognosis. However, in more recent larger-scale studies, many authors consider the safety profile of post-low-voltage injury to be high, and patients can be safely discharged with an initial normal ECG, and arrhythmias are often short-lived and insignificant (Pawlik et al., 2016; Pilecky et al., 2019). This recommendation is reflected within the current Joint Royal Colleges Ambulance Liaison Committee (JRCALC) guidelines (Association of Ambulance Chief Executives, 2021); however, interestingly there is currently no other national guidance within the United Kingdom. There is also no inclusion of the previously published ‘electrocution’ chapter within the current European Resuscitation Council guidelines (Soar et al., 2021). This arguably may have contributed to inconsistencies in how individual emergency departments (EDs) manage these patients, with some patients being discharged immediately, and some admitted for up to 24 hours (Warentis et al., 2020). Therefore, clinicians often base their decisions on clinical opinion or traditionally led thinking, rather than an evidence-based approach.

Therefore, to better inform paramedic practice for clinicians in acute healthcare settings, this literature review aims to determine:

1. if there are any common arrhythmias, and the frequency that they may occur post-low-voltage electrical injury;
2. if any arrhythmias may require intervention or treatment; and
3. the safety profile of discharging a patient post voltage electrical injury with an apparently normal ECG in an acute healthcare setting, and the occurrence of the ‘delayed arrhythmia’.

## Methods

Throughout the review process, the ‘preferred reporting items for systematic reviews and meta-analyses’ (PRISMA) guidelines were used to assist and refine the search process to ensure the articles were relevant to the review’s topic and focus (Moher et al., 2015). The search was conducted in January 2022 on the MEDLINE, CINAHL and AMED databases to identify papers in English published between the period of 1980–2022. The year 1980 was chosen as, although studies from this date may be considered dated and there have been significant developments in healthcare during this time frame, some studies were still considered to be relevant to the review objectives. The identification of arrhythmias post low-voltage electrical injury still remains the same, with the arguable difference of improved technological advances in ECG monitors to increase the sensitivity of detection. However, some of the older studies also provided useful information, as patients with a ‘normal’ ECG were routinely admitted for longer periods of cardiac monitoring, rather than being immediately discharged, as with current practice.

The search terms used were as follows: (‘electric* injury’ OR electrocution) AND (ECG or electrocardiogram) AND (arrhythmia OR dysrhythmia OR prognosis).

Prior to publication, a further search was conducted in April 2023 to ensure no further relevant studies had been published (see Table 1).

**Table 1.** Inclusion and exclusion criteria.

Inclusion criteria	Exclusion criteria
Worldwide studies	
Studies written in English	
Study participants aged 18 years and over	
Peer-reviewed journal articles with primary data	Consensus papers, unpublished papers, internet articles/discussions, guidelines, single case studies, systematic reviews
Low-voltage electric shocks < 1000 V	Studies involving police TASER weapons
Publications between 1980 and 2022	
Studies focusing on the cardiovascular/ECG effects of electrical injury	Studies concentrating solely on the treatment or prognosis of thermal/electrical burns, injuries such as fractures, psychiatric outcomes, blood biomarkers

ECG: electrocardiogram.

Particular attention was paid to what voltage many studies considered ‘low’, as < 1000 V is not a globally recognised standard. Studies that also contained high-voltage patients were included, but only those with clear separation of the data from the low-voltage patient groups. Studies involving police TASER devices were also excluded, as it is likely patients may have other factors involved at the time of injury, including extreme stress, physical exertion or being under the influence of alcohol or drugs (Zipes, 2014).

**Figure 1:**
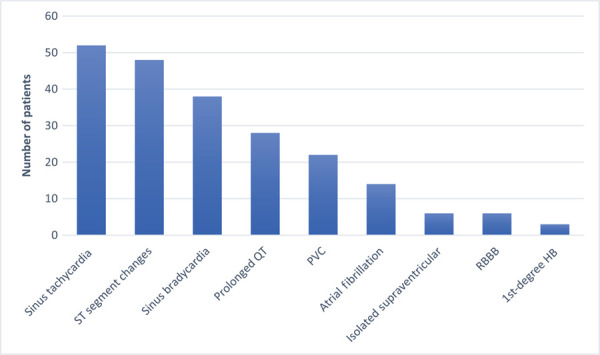
Figure 1. PRISMA flow diagram of search results (Moher et al., 2015).

The databases were searched within the platform EBSCOhost, where duplicates were removed (by SC). The title and abstract were screened for relevance, whereby articles which were clearly not relevant were removed (by SC). The full text was examined for relevance against the eligibility criteria (by SC), and this was checked (by SL and AR). Data pertaining to the objectives were extracted from the articles (by SC) and checked (by SL and AR). (For the PRISMA flow diagram, see Figure 1.)

The quality of the studies was assessed (by SC) using the Critical Appraisal Skills Programme (CASP) checklist (Critical Appraisal Skills Programme, 2018) to formulate opinions and conclusions about each study while reducing bias (Long et al., 2020). This method also provided structure and consistency, while increasing the reliability and validity of the opinions of the reviewer (Centre for Reviews and Dissemination, 2009). However, as many studies were retrospective reviews, the CASP checklist for ‘cohort studies’ was used but with modifications made to allow for the lack of an ‘intervention’. Each study was therefore reviewed using the same modified CASP checklist to provide a consistent approach and reduce bias (Long et al., 2020). Finally, a descriptive statistics approach was used to quantitively describe and summarise the final data (Kaur et al., 2018).

## Results

**Figure 2:**
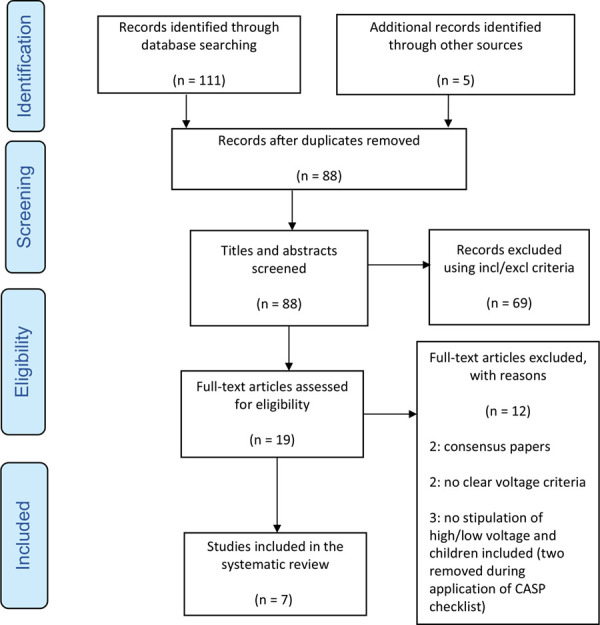
Figure 2. Summary of arrhythmias.

AF: atrial fibrillation; HB: heart block; PVC: premature ventricular contractions; RBBB: right bundle branch block.

Two studies (Bailey et al., 2007; Pilecky et al., 2019) were removed when the CASP checklist was applied (and appear in the PRISMA flow diagram; Figure 1) as it was not clear how the patients had been separated into groups according to the voltages. The other seven studies were retained and included in this study as they were deemed to be of sufficient quality. The seven studies are detailed in Table 2. From the accumulated data of the seven studies, 26% of patients were observed to have an abnormal ECG feature (n = 346/1234), with the commonest including sinus tachycardia, ST segment changes and sinus bradycardia (see Figure 2). Only one study identified a small minority of patients (n = 6) who required treatment with atropine drug therapy to correct a sinus bradycardia with inadequate perfusion. All patients who had an initial arrhythmia on arrival to the ED were monitored between 6 and 48 hours, and only one study recorded three deaths but due to additional complications of sepsis or associated trauma. There were no recorded cases of delayed arrhythmias throughout. A consensus across all the studies was the safety of discharging a patient immediately following an initial, normal ECG. See Tables 2 and 3 for an accurate summary of the studies’ characteristics and data.

**Table 2.** Study characteristics.

Study	Study type	Country	Study methods
Dhital et al. (2020)	Observational prospective study	Nepal	High- and low-voltage electrical injury patients on attendance to ED received an initial ECG, then admitted to a cardiac unit for 48 hours. All patients received 1500 ml of intravenous 0.9% saline fluids.
Searle et al. (2013)	Retrospective analysis	Germany	Review of adult and paediatric patients with high-low voltage electrical injuries with a main focus of the evaluation of delayed arrhythmias. All patients had a standardised period of 12 hours cardiac monitoring.
Blackwell & Hayllar (2002)	Prospective observational study	Australia	The study focused on low-voltage electrical injuries and admitted all patients for a period of 6 hours of cardiac monitoring. Any persistent arrhythmias patient were admitted or if ‘normal’ the patient was discharged.
Warentis et al. (2020)	Retrospective analysis	Germany/Austria	High- and low-voltage electrical injuries were reviewed in attendance to two ED units. The study admitted the patients to examine not only the ECG findings but also the associated symptoms and injury characteristics.
Ahmed et al. (2021)	Retrospective chart review	Norway	All adult patients with both high- and low-voltage electrical injuries were examined with a focus on the ECG features, safety of discharge and the overall 30-day mortality.
Akkas et al. (2012)	Retrospective chart review	Turkey	Examination of high- and low-voltage electrical injury patients attending an ED. The data also included patient demographics and time from injury to ECG, however the study had no standardised period of cardiac monitoring.
Arrowsmith et al. (1997)	Retrospective review	United Kingdom	Examination of electrical injury patients from a UK burns unit and the presenting ECG features. Most patients were monitored for a period of 24 hours.

ED: emergency department; ECG: electrocardiogram.

**Table 3.** Study results.

Study	Sample size (low voltage)	Stated low voltage range	No. pts with ‘normal’ ECG	No. pts discharged on initial assessment	No. pts with initial arrhythmia	Initial cardiac monitoring period (hrs)	No. pts with resolution of arrhythmia	No. pts admitted post initial monitoring period	No. pts with delayed arrhythmia	No. pts requiring treatment or intervention	Mortality (%)
Dhital et al. (2020)	32	< 1000 V	88% (n = 28)	88% (n = 28)	12% (n = 4)	48.0	100% (n = 4)	0	0	0	0
Searle et al. (2013)	153	< 500 V	82% (n = 126)	*	18% (n = 27)	20.3	*	*	0	0	0
Blackwell & Hayllar (2002)	196	< 1000 V	59% (n = 116)	3% (n = 6)	38% (n = 74)	6.0	*	4% (n = 8)	0	3% (n = 6)	0
Warentis et al. (2020)	167	25–980 V	10% (n = 17)	0	14% (n = 24)	24.0	12% (n = 20)	2% (n = 4)	0	0	0
Ahmed et al. (2021)	408	< 1000 V	79% (n = 323)	*	21% (n = 85)	*	*	1% (n = 6)	0	0	0
Akkas et al. (2012)	65	< 1000 V	83% (n = 54)	*	9% (n = 6)	48.0	*	*	0	*	5% (n = 3)
Arrowsmith et al. (1997)	128	< 1000 V	99% (n = 127)	0	1% (n = 1)	24.0	1% (n = 1)	1% (n = 1)	0	0	0

*Missing or incomplete data.

ECG: electrocardiogram.

## Discussion

### Objective 1: arrythmias observed post low-voltage electrical injury

Despite the summary of arrhythmias demonstrated in Figure 2, one of the main limitations determined in this review is that no study was able to access a patient’s previous ECGs or detailed past medical history to account for the possibility of a particular ECG feature being present prior to the electrical injury, and therefore to establish a potential ‘control group’. This is a significant shortcoming, as all but one study recorded patients being largely asymptomatic and most arrhythmias remaining until discharge. This therefore means that the arrhythmia could have potentially been a ‘normal’ finding for the patient, such as with right bundle branch block (RBBB) (Alventosa-Zaidin et al., 2019).

Although this limitation was considered only by Warentis et al. (2020) and briefly discussed with individual arrhythmia patients, this issue is arguably unavoidable. It is very common for younger and otherwise healthy adults to not have a previous ECG available, particularly within the United Kingdom as large-scale ECG screening is not undertaken in current practice (Chamley et al., 2019).

It is reasonable to consider that if the spontaneous resolution of an arrhythmia was documented during cardiac monitoring, this may be the only indication if it was likely to have been provoked by an electrical injury. However, only three of the seven included studies provided a sufficient level of detail to clarify if an arrhythmia subsequently resolved during the cardiac monitoring period (Arrowsmith et al., 1997; Dhital et al., 2020; Warentis et al., 2020). For example, in the Arrowsmith et al. (1997) study the author discussed two cases where premature ventricular contractions were seen on initial presentation but were observed to disappear after 24 and 48 hours. This issue may also be compounded by the difference in standardised monitoring periods throughout the studies that ranged from 6 to 48 hours.

A second limitation overlooked in five of the review studies is that only two studies considered that the presenting arrhythmia may be attributed to causes other than the electrical injury (Ahmed et al., 2021; Warentis et al., 2020). For example, sinus tachycardia was one of the most common ECG features observed throughout the studies, however this may not be thought of as an arrhythmia at all, as it is instigated by physiological causes such as pain, stress or anxiety (Orme & Channer, 1999; Soar et al., 2021; Yusuf & Camm, 2005). These are common symptoms experienced post electrical injury, even from a lower-voltage source (Biering et al., 2021). Similarly, with young and athletic patients, sinus bradycardia may also be a normal variant due to a higher level of cardiovascular fitness or patients taking beta blocker drugs (Bessem et al., 2018; Hafeez & Grossman, 2020). However, only two studies within the review recorded patients’ past medical history and prescription medications (Dhital et al., 2020; Searle et al., 2013).

Lastly, the importance of obtaining a 12-lead ECG post electrical injury has been considered important due to the vulnerability of the right coronary artery because of its proximity to the chest surface in electrical shocks, and the potential risk of coronary artery spasm or myocardial damage (Guinard et al., 1987). However, another notable issue with the studies within the review was the irregular, or even lack of, reporting of 12-lead ECGs. An example of this was with the Blackwell and Hayllar (2002) study whereby only 26% of patients had a second 12-lead ECG recorded to compare any changes from the initial ECG prior to discharge. However, interestingly, in some of the studies within this review no reference was made to obtaining a pre-cordial lead ECG at all (Akkas et al., 2012; Arrowsmith et al., 1997; Dhital et al., 2020).

Comparative studies of lower-voltage electrical injury patients that also included a small high-voltage group include Pawlik et al. (2016), which retrospectively reviewed 234 cases of electrocution from a 10-year period in a large ED. Only four patients were observed to suffer arrhythmias, including sinus bradycardia, two premature ventricular beats and an atrial fibrillation (AF). No late onset arrhythmias were recorded and all the patients survived requiring no treatment to manage the initial arrhythmias. Similarly, Pilecky et al. (2019) reviewed the ECGs of 480 patients retrospectively between 2011 and 2016 post electrocution, with the majority occurring from a low-voltage source. The most frequent arrhythmias included sinus bradycardia (n = 50, 10.4%) and sinus tachycardia (n = 21, 4.4%).

A recent retrospective review study not included in the review series due to limited patient ECG data was a Norwegian study that evaluated the symptoms, ECG features and blood biomarkers of low-voltage electrical injury patients attending an ED between 2012 and 2017 (Svendsen et al., 2022). Similar to our study, 20% of patients (n = 41/209) were found to have an abnormal ECG on initial assessment; arrhythmias such as ventricular extrasystoles, RBBB, ST elevation changes and a single case of second-degree AV block (Mobitz type 1) were recorded. The study was limited as it did not detail specific patient numbers with arrhythmias or cardiac monitoring periods. However, also comparable to our review, the authors commented on the challenge of concluding if the presenting arrhythmias could be attributed to the electrical shock, as only 73% (n = 30/41) of patients had a repeat ECG before discharge, and a further 33% (n = 10/30) were only observed to have a resolution of their arrhythmias prior to discharge.

### Objective 2: arrythmias warranting admission or treatment

Within the review series, a minority of notable arrhythmias were apparent. Blackwell and Hayllar (2002) identified 28 patients with long QT syndrome. In all but three patients, it resolved within 6 hours spontaneously without intervention, however no further information was detailed on the treatment or length of admission that was required for the remaining three patients. A QT prolongation is associated with a high risk of syncope, seizures and sudden cardiac arrest. While long QT syndrome is often a congenital abnormality, it can also be ‘acquired’ in cases such as hypoelectrolytaemia with low potassium or magnesium levels, sometimes seen in chronic alcoholism or gastrointestinal losses (Moss & Kass, 2005). This arrhythmia could therefore be considered a potentially significant finding, but the research is unclear on the prognosis of QT prolongation post electrical injury when compared to ‘native’ QT prolongation. Only one other dated case report of QT prolongation associated with an electrical injury has previously been documented, however this was due to a high-voltage lightning injury (Palmer, 1987).

The second potentially significant arrhythmia was AF that was observed in three studies, albeit in very few cases (Ahmed et al., 2021; Dhital et al., 2020; Warentis et al., 2020). The association and long-term prognosis of patients experiencing AF post electrical injury is unknown. Within this case series, some authors commented on the self-limiting nature of AF after a low-voltage injury, but no study author considered the possibility of a biphasic presentation of AF to therefore warrant longer-term evaluation of the patient. Only single case reports of AF post low-voltage electrical injury have been published, but there is considerable variance in the patient outcomes, with some patients requiring anti-arrhythmic drugs or cardioversion (Akdemir et al., 2004; Bilik et al., 2016; Ghosh et al., 2021), while other patients experienced spontaneous resolution of the arrhythmia without treatment (Biswas et al., 2015; Duchini et al., 2018). However, no research was found into the longer-term prognosis of an AF patient post electrical injury.

‘ST-T segment changes’ were described in four of the studies, ambiguously as ‘insignificant’. However, the reason for this determination was likely due to all of the studies having access to troponin blood markers as an additional assessment to accompany the 12-lead ECG, to make a more definitive determination. Blood analysis is not always available in other acute healthcare areas such as urgent treatment centres or ambulances, and therefore arguably may not always be possible to conclude so easily within these settings. The association with the pre-cordial lead changes and low-voltage injury is lacking, as much of the past evidence is taken from high-voltage or lightning injury case reports with ST segment changes (Vianello, 1997; Yildiz, 2018).

It is widely considered in the literature that clinically significant arrhythmias post low-voltage injury are rare and often self-limiting, requiring no intervention or treatment (Bailey et al., 2007; Svendsen et al., 2022). However, this theory could be challenged, as the Blackwell and Hayllar (2002) study observed six patients who required intravenous atropine drug therapy for atrioventricular blocks due to significant bradycardias and hypotension, but with rapid resolution of symptoms post treatment. Unfortunately, the study presented limited detail such as how long the patients were admitted for, if they required cardiology follow-up or if there was a requirement for longer-term medication post discharge. The Akkas et al. (2012) study was the only study to observe mortality from a low-voltage source. However, although limited detail is provided regarding the mechanism of injury, the author does reference that there was exposure to ‘severe trauma and sepsis’, indicating atypical injury circumstances. It is arguable that many cases of significant cardiac arrhythmias or deaths from voltage injury can be attributed to other factors such as conduction methods such as water involvement, transthoracic current passage or if a patient was exposed to an alternating current (Warentis et al., 2020).

### Objective 3: safety profile of discharging a patient with a normal electrocardiogram and the ‘delayed arrhythmia’

Five studies discharged patients immediately following a ‘normal’ ECG, and therefore concluded that there was a high safety margin for adopting this as a standard of practice. However, it is a significant oversight and limitation that after discharge, little to no further evaluation of the patient was completed. In the studies that did consider a post-discharge review, this was mostly limited to a ‘30-day mortality’ assessment using the patients’ electronic medical records. While this is arguably some limited indication of the short-term outcome of the patient, this method is severely lacking, as all but one of the studies recorded whether a patient re-attended the same ED at a later date. This limitation may largely be due to the constraints of a retrospective review’s set ethical and legal parameters on communication with patients (Tofthagen, 2012), however no study evaluated whether a patient attended a different acute healthcare setting or required further assessment or treatment with their own GP. As past authors have raised concern of the possibility of delayed and significant arrhythmias occurring up to three months post discharge (Jensen et al., 1987), there is also a larger question as to whether morbidity or mortality occurred after the 30-day review period.

Of the studies reviewed in detail, only three sought to monitor patients within the ED for a set period, with a patient presenting with a ‘normal’ ECG in an effort to investigate the possibility of the ‘delayed arrhythmia’ (Akkas et al., 2012; Arrowsmith et al., 1997; Searle et al., 2013). However, there was considerable variance of the period of cardiac monitoring which ranged from 6 to 48 hours. Although it should be noted that no onset of a life-threatening or significant ‘delayed arrhythmia’ occurred in any of the studies during the stated cardiac monitoring periods, it could still be argued that even consolidated, these studies contain limited patient numbers, varied patient demographics and varied timing of presentation of the patient from the electrical injury to attending the hospital.

However, a more recent Danish study reviewed 11,462 high and low electrical patients admitted from 1994 to 2011, and found that no patient experienced a late onset arrhythmia (Hansen et al., 2017). In a study of 134 patients that attended the ED post transthoracic current exposure, 11% suffered an initial abnormal ECG, with no patient requiring treatment, and no delayed arrhythmias being observed over a 24-hour standardised monitoring period (Bailey et al., 2007).

### Limitations

Across the review studies, only one study included pre-hospital data obtained by ambulance healthcare professionals, which may arguably exclude a wider range of data. Similarly, there was also limited record keeping from the time of injury until when a patient presented to the ED, patient demographics or patient past medical history that may increase their susceptibility to cardiac injury. Many of the studies included within the review are limited by small sample sizes, from single-centre ED departments. This review also did not review electrical injuries and children (< 18 years old) or the use of cardiac biomarkers.

## Conclusions

It is possible that there are common arrhythmias that may be potentially associated with low-voltage electrical injuries. Paramedics are placed in a unique position to be able to identify these arrhythmias at an earlier stage, and this may provide clinically important data for determining a patient disposition. While there is some suggestion most arrhythmias are self-limiting and seldom require treatment, particular attention must be paid to those arrhythmias that may be potentially more clinically significant, such as prolonged QT, or bradycardias that require intervention. Should arrhythmias be apparent on initial assessment, paramedics should consider ongoing cardiac monitoring throughout the entirety of the patient consultation or conveyance to hospital, and be prepared to respond to any clinical deterioration.

From the limited number of patients involved in this review, there is some suggestion that the entity of the ‘delayed arrhythmia’ may not be a viable reason for patients to be admitted for extended periods. This may also mean that the normal ECG post low-voltage injury may be a useful indicator of safely discharging from the initial assessment, which supports the current JRCALC guidance. However, the evidence for this is weak, and further studies should aim to consider the longer-term outcomes of this patient group before a definitive conclusion can be reached. Should a patient be appropriate for discharge with a normal ECG, paramedics should recognise that the ECG forms one element of a global assessment of the patient, and the importance of good safety-netting advice and clinical documentation cannot be overstated.

## Author contributions

SC undertook all of the research and wrote the article. It was primarily completed as part of SC’s dissertation for the MSc Healthcare Practice, but was re-written for the article. SL and AR were SC’s tutors during the dissertation module at St. George’s, University of London. They helped to write the original piece of work, and gave guidance and feedback on various drafts. SC acts as the guarantor for this article.

## Conflict of interest

None declared.

## Ethics

Not required.

## Funding

None.
